# The Effect of Xanthohumol and Thymol on *Candida albicans* Filamentation and Its Impact on the Structure, Size, and Cell Viability of Biofilms Developed over Implant Surfaces

**DOI:** 10.3390/cells13221877

**Published:** 2024-11-13

**Authors:** Enrique Bravo, Marion Arce, David Herrera, Mariano Sanz

**Affiliations:** 1 ETEP (Etiology and Therapy of Periodontal and Peri-Implant Diseases) Research Group, Faculty of Dentistry, Complutense University, 28040 Madrid, Spain; ebravofe@ucm.es (E.B.); davidher@odon.ucm.es (D.H.); 2 Department of Conservative Dentistry, Faculty of Dentistry, University of Chile, Santiago 8380544, Chile; m.arce@odontologia.uchile.cl

**Keywords:** peri-implantitis, oral biofilm, *Candida albicans*, xanthohumol, thymol

## Abstract

The aim of this in vitro study was to evaluate the effect of xanthohumol and thymol on the impact of *Candida albicans* on the structure, size and cell viability of subgingival biofilms formed on dental implant surfaces. The structure and microbial biomass of biofilms developed after 72 h, treated and untreated with both extracts, were compared by scanning electron microscopy (SEM) and confocal laser microscopy (CLSM). Quantitative polymerase chain reaction (qPCR) was used to quantify the number of viable and total microorganisms of each of the biofilm-forming strains in each condition. A general linear model was used to compare and validate the CLSM and qPCR results. The presence of xanthohumol and thymol during biofilm development inhibited the filamentous growth of *C. albicans*. The biofilm incubated with xanthohumol had significantly lower bacterial biomass and cell viability than the biofilm not exposed to the extract (*p* < 0.05). In contrast, these global parameters showed no differences when the biofilm was incubated with thymol. In the presence of xanthohumol, there was a decrease in counts and cell viability of *Fusobacterium nucleatum*, *Porphyromonas gingivalis*, and *Aggregatibacter actinomycetemcomitans*. Thymol treatment reduced the viability of *F. nucleatum* and *P. gingivalis*. The presence of these vegetable extracts during the development of a dynamic in vitro multispecies biofilm model inhibited the filamentous growth of *C. albicans*, partially reversing the effect that the fungus exerted on the structure, size and vitality of periodontopathogenic bacteria.

## 1. Introduction

Dental implants are a widely accepted tool to rehabilitate total or partial edulism [1,2], having demonstrated high long-term success rates, although with a high incidence of complications, mainly peri-implant diseases. In the World Workshop on the Classification of Periodontal and Peri-implant Diseases and Conditions (2017), these diseases were classified as peri-implant mucositis and peri-implantitis, with prevalences ranging from 43 to 47% and 20 to 22%, respectively [3]. Both are inflammatory conditions resulting from the accumulation of biofilms on the surface of dental implants or the associated prosthetic components, which, in the case of peri-implantitis, are associated with progressive bone destruction that, if left untreated, may result in implant loss [4,5,6]. These submarginal biofilms consist of microbial communities within an extracellular matrix. The complex polymicrobial communities consist primarily of bacteria but may include viruses, protozoa, and fungi [7,8,9,10]. In the case of biofilms, composed of both bacteria and fungi, complex inter-kingdom relationships can be established, either favoring bacterial colonization or increasing the intrinsic virulence of these biofilms [11,12,13].

*Candida albicans*, a facultative dimorphic anaerobic fungus, is the most frequent eukaryotic biofilm-forming pathogenic microorganism in the oral cavity [14]. Although the data from clinical studies are heterogeneous, there is a consistently higher prevalence of *C. albicans* in biofilms from subjects with periodontitis than those from subjects with good periodontal health [15]. Similarly, the few studies evaluating the prevalence of *C. albicans* in peri-implant diseases have reported a higher prevalence in patients with peri-implantitis [16,17,18,19].

The pathogenicity of *C. albicans* is mainly related to its filamentous growth. The development of pseudohyphae is associated with the secretion of hydrolytic enzymes, basically lipases, proteases, and hemolysins, that trigger the chronic inflammatory response associated with the infected tissue [20,21], which, if unresolved, may lead to the alveolar bone resorption characteristic of peri-implantitis [22,23]. A predominance of Gram-negative bacteria associated with the anaerobic environment and nutrient selection associated with the submarginal pocket conditions favors *C. albicans* growth through hyphae to the detriment of yeast growth [24]. A recent study from our research group has demonstrated that *C. albicans* growth through pseudohyphae favors the anchorage of bacteria during biofilm maturation, increasing their viability and progression on dental implant surfaces [25]. Furthermore, several in vitro studies have demonstrated the association between fungus and periodontopathogenic bacteria, leading to dysbiotic biofilms and increasing their virulence [11,26,27,28,29,30,31,32].

Therefore, therapies aimed at inhibiting or blocking *C. albicans* filamentation may facilitate the onset and/or progression of periodontal and peri-implant diseases. In this context, so-called phytochemicals (natural substances of plant origin) exhibiting inhibitory properties in the process of *C. albicans* filamentation could be considered antifungal agents. These extracts have also shown activity against periodontopathogenic bacteria [33,34,35].

**Figure 1:**
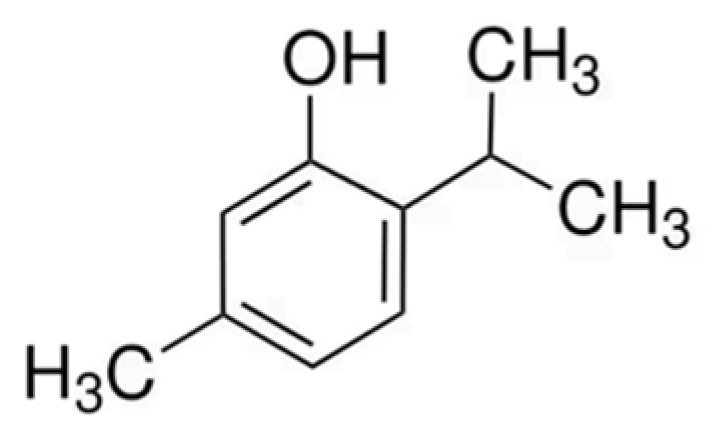
Chemical structure of thymol.

One of these phytochemicals of interest is thymol, an aromatic polyphenol (C_10_H_14_O) (Figure 1) found in the leaves of plants such as oregano (*Origanum vulgare*), thyme (*Thymus vulgaris*) or basil (*Ocimum basilicum*). Its mechanism of action includes the suppression of cellulase and peptinase enzyme activities, linked to the hyphal formation process, thus preventing the filamentous growth of the fungi [36,37].

**Figure 2:**
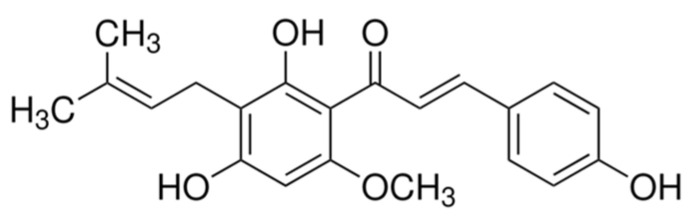
Chemical structure of xanthohumol.

A second phytochemical of interest is xanthohumol, a prenylated flavonoid (C_21_H_22_O_5_) (Figure 2) from the female flowers of the hop plant (*Humulus lupulus*) that exhibits antiseptic, anti-inflammatory, antidiuretic, and antioxidant properties [38]. The mechanism of action of xanthohumol is related to its inhibition of quorum sensing and de-stabilization of the microbial cell wall [39], with demonstrated activity against *C. albicans* biofilms by limiting their ability to adhere and form filaments [40,41].

Thus, this in vitro investigation aimed to determine whether inhibition of *C. albicans* pseudohyphal growth using thymol and xanthohumol would impact biofilm growth and pathogenicity.

## 2. Materials and Methods

### 2.1. Microbial Strains and Culture Conditions

Bacterial strains Streptococcus oralis CECT 907T, Actinomyces naeslundii ATCC 19039, Veillonella parvula NCTC 11810, Fusobacterium nucleatum DMSZ 20482, Porphyromonas gingivalis ATCC 33277 and Aggregatibacter actinomycetemcomitans DSMZ 8324 were grown on blood agar plates (Blood Agar Oxoid No 2; Oxoid, Basingstoke, UK), supplemented with 5% (*v*/*v*) sterile horse blood (Oxoid), 5.0 mg/L haemin (Sigma, St. Louis, MO, USA) and 1.0 mg/L menadione (Merck, Darmstadt, Germany) at 37 °C for 24–72 h under anaerobic conditions (10% H_2_, 10% CO_2_ and N_2_ balance). The fungal strain Candida albicans SC 5314 was grown on yeast-peptone-glucose (YPD) agar plates (2% glucose (Panreac, Barcelona, Spain), 2% peptone (Life Technologies, Detroit, MI, USA), 1% peptone yeast extract (Life Technologies, Detroit, MI, USA) and 2% agar (Becton, Dickinson and Company, Sparks, MD, USA) at 37 °C for 24 h under aerobic conditions.

### 2.2. Minimum Inhibitory Concentration (MIC) of Xanthohumol and Thymol Against the Microorganisms Included in the Biofilm Model

To select sublethal concentrations of thymol and xanthohumol for their application in the biofilm model, MICs against each bacterium and *C. albicans* were determined [42].

Isolated colonies of each microorganism were grown in a protein-enriched BHI-modified medium adjusted to pH 7.2 (Becton, Dickinson and Company, Franklin Lakes, NJ, USA), which was supplemented with 2.5 g/L mucin (Oxoid), 1.0 g/L yeast extract (Oxoid), 0.1 g/L cysteine (Sigma), 2.0 g/L sodium bicarbonate (Merck), 5.0 mg/L haemin (Sigma), 1.0 mg/L menadione (Merck) and 0.25% (*v*/*v*) glutamic acid (Sigma) at 37 °C under anaerobic conditions (10% H_2_, 10% CO_2_ and N_2_ equilibrium). Isolated *C. albicans* colonies were grown in a liquid YPD medium under aerobic conditions at 37 °C.

The exponential growth phase was detected spectrophotometrically, with cultures always below an optical density (OD_550 nm_) of 1.2. Once exponential growth was reached, 200 μL of each inoculum was transferred to a 24-well microplate, reaching a final concentration of 10^6^ colony-forming units (CFU)/mL. Then, xanthohumol (NATECO^®^ GmbH & Co, Wolnzach, Germany) and thymol (Sigma-Aldrich^®^, Steinheim, Germany) were added at final concentrations of 6.25, 12.5, 25, 50, 100, 250, 500, and 1000 µM in both cases, using phosphate-buffer saline (PBS) as a negative control. These microplates were incubated for 24 h at 37 °C under anaerobic conditions. The MICs of each extract were determined on blood agar plates for the bacteria and YPD plates for *C. albicans*, on which 100 µL of each suspension was seeded. The plates were incubated for 72 h at 37 °C under anaerobic conditions. The lowest concentrations of thymol and xanthohumol extracts showing visible inhibition of microbial growth were considered the MICs of each extract for each strain, thus selecting a lower concentration range to ensure no effect on cell viability.

All tests were carried out in triplicate, with the respective controls for contamination.

### 2.3. Agar Invasive Assays of C. albicans in the Presence of Xanthohumol and Thymol

To evaluate the inhibitory effect of thymol and xanthohumol on the pseudohyphal growth of *C. albicans*, agar invasiveness assays were performed in the presence of both substances [43].

Isolated *C. albicans* colonies were grown in a liquid YPD medium for 24 h at 37 °C under aerobic conditions. The exponential growth phase was detected spectrophotometrically, with the culture always below an optical density (OD_550 nm_) of 1.2. Once this exponential growth was reached, 10 μL droplets were plated at an optical density equivalent to 10^4^ CFU/mL on YPG agar plates (2% galactose (Panreac, Barcelona, Spain), 2% peptone (Life Technologies, Detroit, MI, USA), 1% peptone yeast extract (Life Technologies, Detroit, MI, USA) and 2% agar (Becton, Dickinson and Company, Sparks, MD, USA). The absence of glucose and the presence of galactose in this growth medium stimulated filamentous growth of *C. albicans* [44]. To evaluate the inhibitory effect on filamentation, these culture plates that contained xanthohumol and thymol at concentrations of 0, 6.25, 12.5, 25, 50 and 100 µM and 0, 62.5, 125, 250, 500 and 1000 µM, respectively, were grown at 37 °C for 48 h under aerobic conditions. After incubation, the inoculation sites were washed by exposing the plates to dripping tap water, and the invasiveness of the culture grown on the agar was assessed in each case. The minimum concentration of each extract at which a reduction in *C. albicans* invasion into the agar was observed was established as optimal for use in the biofilm model.

Tests were carried out in triplicate.

### 2.4. In Vitro Dynamic Multispecies Biofilm Model

A previously validated multispecies dynamic biofilm in vitro model was applied on dental implant surfaces [35,45]. The system consists of a sterile vessel, where the modified BHI medium flows into the bioreactor using a peristaltic pump. The bioreactor (Lambda Minifor© bioreactor, LAMBDA Laboratory Instruments, Sihlbruggstrasse, Switzerland) maintained the culture medium in the selected stable conditions of the oral cavity (temperature of 37 °C, a pH of 7.2, and an anaerobic atmosphere (10% H_2_, 10% CO_2_ and N_2_ balance).

To prepare the bacterial and fungal pre-inoculum, a pure culture of each strain was grown for 24 h under the conditions described above. Microbial growth was measured spectrophotometrically, and a microbial mixture of 10^6^ colony-forming units (CFU)/mL of each bacterium and 10^4^ CFU/mL of *C. albicans* was obtained.

The vessel was inoculated with 5 mL of the microbial mixture and incubated for 12 h under the described conditions. When the multispecies culture grew to the exponential phase, a second peristaltic pump was activated, with a constant flow rate of 30 mL/h, to start continuous culture and transfer the culture to the Robbins device, inside which the dental implants on which the biofilm developed were placed (Straumann^®^ Tissue Level Standard, Straumann Institute AG, Basel, Switzerland). Implant size was 8 mm in length and 3.3 mm in diameter, with the patented SLA surface: moderately rough, sand-sprayed and acid-etched surface. They were kept for 72 h under the conditions described in the interior of the Robbins device until the formation of mature biofilms.

To evaluate the effect of thymol and xanthohumol on the growth and filamentation of *C. albicans* and, consequently, on sub-marginal biofilm formation, 72 h mature biofilms were developed under the following conditions:

- Negative control: biofilms were developed for 72 h using the six bacterial strains of the model.
- Positive control: the six bacterial strains of the model and *C. albicans* included in biofilms for 72 h to generate the dysbiotic event.
- Xanthohumol treatment: The six bacterial strains model and *C. albicans* were included in biofilms for 72 h. Xanthohumol was injected (12.5 µM) into the Robbins device at 12 h intervals.
- Thymol treatment: the six-bacterial-strain model and *C. albicans were* included in biofilms for 72 h. Thymol was injected (250 µM) into the Robbins device at 12 h intervals.

For each mentioned condition, three implants were analyzed by scanning electron microscopy (SEM) (*n* = 3), three by confocal laser scanning microscopy (CLSM) (*n* = 3), and nine by real-time quantitative polymerase chain reaction (qPCR) (*n* = 9).

### 2.5. Scanning Electron Microscopy (SEM)

Following the optimized protocol for analyzing biofilm samples by SEM [45], the implants were removed from the Robbins device and were washed, dried, and coated with gold before analysis. The samples were analyzed using a JSM 6400 electron microscope (JSM6400, JEOL, Tokyo, Japan).

This analysis was conducted at the National Centre of Electron Microscopy (In-stalación Científico-Técnico singular; ICTS) at the Moncloa Campus of the Complutense University of Madrid (Madrid, Spain).

### 2.6. Confocal Laser Scanning Microscopy (CSLM)

Samples were extracted, washed, and stained using the LIVE/DEAD^®^ BacLightTM bacterial viability kit solution and 3% Calcofluor White for bacteria and *C. albicans*, respectively, following the protocols reported by Blanc et al. and Bravo et al. [25,45].

A Leica^®^ LCS SP8 STED 3X inverted microscope (Mannheim, Germany) was used for noninvasive confocal imaging of biofilms. The COMSTAT 2.1 software (www.comstat.dk (accessed on April, May, June and July 2024)) was used to calculate the biomass in micrometres^3^/micrometres^2^ (µm^3^/µm^2^) and the roughness coefficient (Ra*) of the CLSM acquisitions.

This analysis was performed at the Biological Research Centre Margarita Salas (Centro de Investigaciones Biológicas, Consejo Superior de Investigaciones Científicas—CIB-CSIC), located at the Moncloa Campus of the Complutense University of Madrid (Madrid, Spain).

### 2.7. Quantitative Polymerase Chain Reaction (qPCR)

Samples were extracted, washed, and disaggregated from the implants following the method described by Sánchez et al. [46]. Fractions of 200 µL of each sample were dissociated, treating one of them with propidium monoazide (PMA) to quantify each species’ viability percentage in each condition [25].

DNA was obtained using the commercial kit MolYsisComplete5 (Molzym GmbH & CoKG, Bremen, Germany), following the manufacturer’s instructions (the protocol for microbial DNA extraction will be followed from step 6, avoiding preliminary steps). Primers and probes selected were provided by Life Technologies Invitrogen (Carlsbad, CA, USA), Applied Biosystems (Carlsbad, CA, USA), and Roche (Roche Diagnostic GmbH, Mann-heim, Germany).

The amplification reaction was performed in a total mix volume of 10 μL. Reaction mixtures included 5 μL of MasterMix 2x (LC 480 Probes Master, Roche), optimal primer and probe concentrations, and 2.5 μL of DNA extracted from the samples. The negative control was 2.5 μL of sterile water (non-template control (NTC)) (Roche) [45,47].

The amplification program used included an initial cycle at 95 °C for 10 min, followed by 40 cycles at 95 °C for 15 s and 60 °C for 1 min in a LightCycler^®^ 480 II thermal cycler (Roche Diagnostic GmbH, Mannheim, Germany). LightCycler480 Multiwell Plate 384 White microplates (Roche) were used.

Each DNA sample was analyzed in duplicate. The provided software determined the quantification cycle (Cq) values (LC 480 Software 1.5, Roche). For cell quantification, the data reported was extrapolated with previously designed standard curves with the Cq values generated in qPCR vs. log CFU/mL. The software (LC 480 Software 1.5, Roche) automatically generated a correlation between Cq values and CFU/mL.

### 2.8. Statistical Analysis

The primary outcome variable was the counts of viable and total bacteria present in the biofilm of each condition, measured by qPCR and expressed as CFU/mL. The secondary outcome variable was the microbial biomass obtained by CLSM and expressed in µm^3^/µm^2^. Data are shown as means and standard deviations (SDs). The Shapiro–Wilk good-ness-of-fit test was applied to assess data normality. When the two sets of data compared showed a normal distribution, a *T*-test with Welch’s correction was executed. When at least one of the two groups did not show a normal distribution, a Mann–Whitney test was used. Comparisons that reported *p*-values < 0.05 were considered statistically significant.

GraphPad Prism software was used for all data analysis.

## 3. Results

### 3.1. MICs of Thymol and Xanthohumol on Planktonic Microorganisms

For xanthohumol, the MICs were set at 25 µM for *S. oralis*, *V. parvula, A. naeslundii*, and *F. nucleatum*, 50 µM for *P. gingivalis* and *C. albicans*, and 100 µM for *A. actinomycetemcomitans*.

For thymol, the MICs were set at 500 µM for *S. oralis, A. naeslundii, V. parvula, F. nucleatum, A. actinomycetemcomitans*, and *C. albicans*, and 1 mM for *P. gingivalis*.

### 3.2. Agar Invasive Assays of C. albicans in the Presence of Thymol and Xanthohumol

The ability of xanthohumol and thymol to inhibit filamentous growth of *C. albicans* was tested by assessing the fungus’s ability to invade agar at increasing concentrations of both substances.

**Figure 3:**
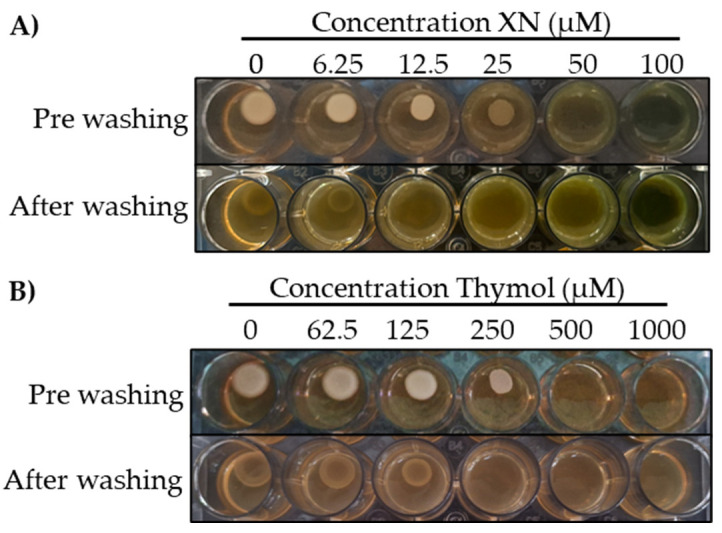
Growth of *Candida albicans* at increasing concentrations of xanthohumol (**A**) and thymol (**B**). Images obtained before washing show superficial yeast growth, and images obtained after washing show filamentous growth into the agar of the same culture.

As shown in Figure 3, the MICs of xanthohumol and thymol on *C. albicans* filamentation were 12.5 and 250 µM, respectively. The surface yeast growth was not altered at these concentrations of both extracts.

### 3.3. SEM Analysis

**Figure 4:**
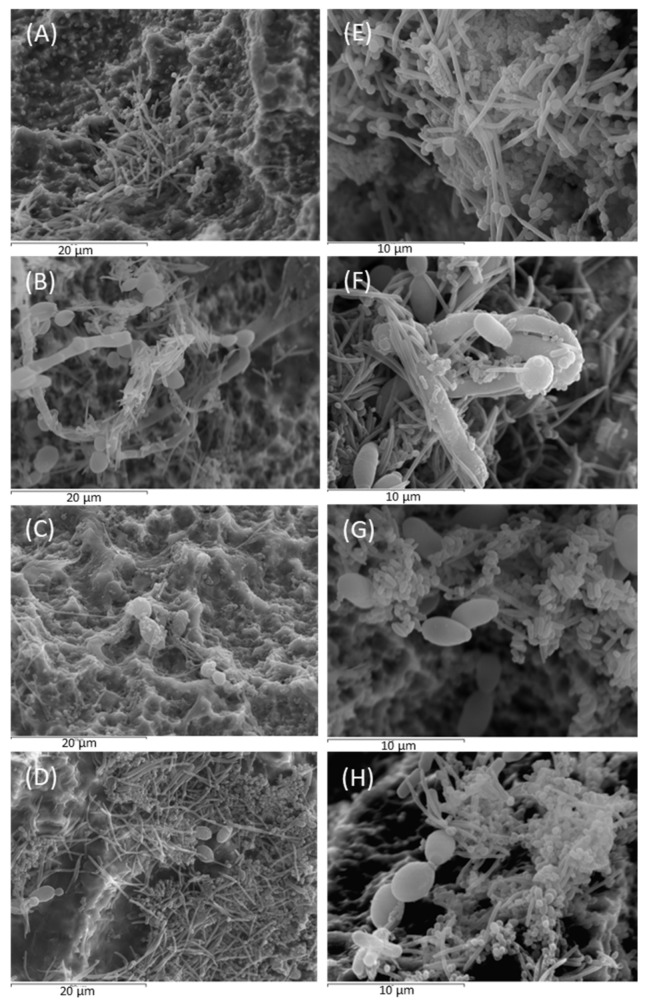
Images obtained by scanning electron microscopy (SEM), with 2500× magnification, of negative control biofilms (in the absence of *Candida albicans*), positive control biofilms (in the presence of *C. albicans*), biofilms developed with *C. albicans* and treated with xanthohumol and biofilms developed with *C. albicans* and treated with thymol ((**A**), (**B**), (**C**) and (**D**), respectively) (scale bar = 20 µm). Figures (**E**–**H**) show the same biofilm clusters with 5000× amplification (scale bar = 10 µm).

Figure 4 shows the biofilms developed after 72 in the presence of *C. albicans* treated with thymol and xanthohumol, without treatment (positive control), and in the absence of fungus (negative control).

*C. albicans* hyphae developed during the biofilm’s maturation resulted in architecture with increased robustness and solidity (Figure 4B). The filaments acted as anchoring support for bacteria, mainly fusiform forms (most probably *F. nucleatum*) and coccobacilli (*P. gingivalis* and *A. actinomycetemcomintans*) (Figure 4F).

Treatments with thymol and, to a greater extent, with xanthohumol prevented the filamentous development of *C. albicans*, resulting in less compact biofilms, where bacterial cells aggregated without the yeast support (Figure 4G,H). In fact, biofilms developed in the presence of the fungus and treated with both extracts (Figure 4C,D) depicted a similar appearance to the mature biofilm without *C. albicans* (Figure 4A,E).

### 3.4. CLSM Analysis

**Figure 5:**
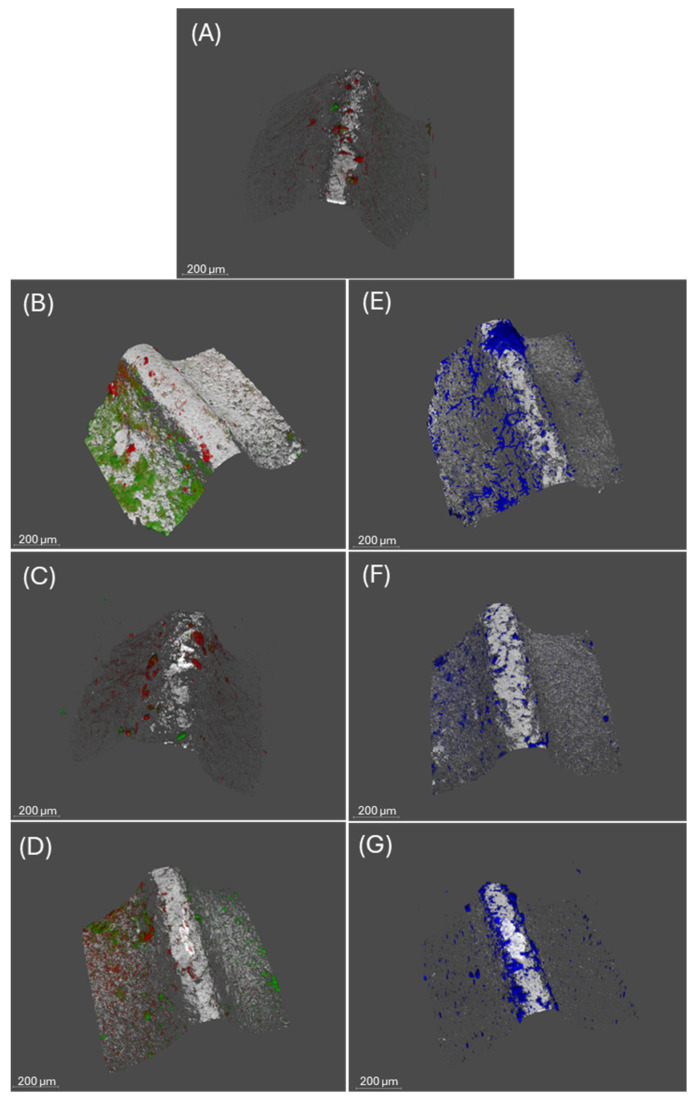
Images obtained with confocal laser scanning microscopy (CLSM) on negative control biofilms (in the absence of *Candida albicans*), positive control biofilms (in the presence of *C. albicans*), biofilms developed with *C. albicans* and treated with xanthohumol and biofilms developed with *C. albicans* and treated with thymol, developed at 72 h ((**A**), (**B**), (**C**) and (**D**), respectively). Images (**E**), (**F**), and (**G**) show *C. albicans* on biofilms developed in the absence and presence of xanthohumol and thymol, respectively. (scale bar = 200 µm). LIVE/DEAD^®^ BackLight Kit was used to stain live bacteria (green), dead bacteria (red), and implant surfaces (white). Calcofluor White (CFW) was used to stain *C. albicans* (blue).

**Figure 6:**
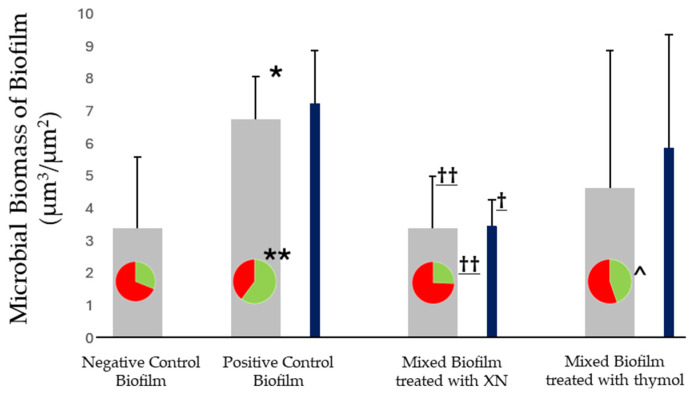
Bacterial biomass (grey bar) and *Candida albicans* biomass (blue bar) [expressed as microbial biomass of biofilm (µm^3^/µm^2^)] of biofilms obtained by quantification of images of confocal laser scanning microscopy (CLSM). The internal cyclogram shows the proportion of viable (green) and dead (red) bacterial cells at each biofilm cluster. * *p* < 0.05 and ** *p* < 0.01: statistically significant differences when comparing negative and positive control biofilms. † *p* < 0.05 and †† *p* < 0.01: statistically significant differences when comparing positive control biofilms with biofilms incubated in the presence of xanthohumol and thymol. ^ *p* < 0.05: statistically significant differences when comparing between biofilms incubated with xanthohumol and thymol.

Figure 5 and Figure 6 show the impact of xanthohumol and thymol treatment on biofilms developed in the presence of *C. albicans*. These mature biofilms, in the presence of *C. albicans* (positive control), demonstrated higher bacterial biomass compared with the negative control biofilms (6.77 µm^3^/µm^2^, SD = 1.32 and 3.39 µm^3^/µm^2^, SD = 2.19; respectively). Furthermore, as shown in Figure 5A,B, the bacterial viabilities of the biofilms developed with and without *C. albicans* were 60.33% (SD = 17.55%), and 31.12% (SD = 12.83%), respectively, demonstrating statistically significant differences (*p* < 0.05). Figure 5E shows the biomass corresponding to *C. albicans*, which was 7.26 µm^3^/µm^2^ (SD = 1.64).

When these mixed biofilms were treated with xanthohumol during their development (Figure 5C), the bacterial density and cell viability were lower compared to non-treated biofilms (3.38 µm^3^/µm^2^, SD = 1.61 and 25.55%, SD = 9.2%; respectively). Similarly, the fungal biomass was significantly lower than the controls, being 3.48 µm^3^/µm^2^ (SD = 0.8), (Figure 5F). The same biofilms incubated with thymol (Figure 5D) demonstrated similar patterns, with reduced bacterial density (4.64 µm^3^/µm^2^, SD = 4.23) and a lower percentage of cell viability (44.63%, SD = 12.91%) when compared with the untreated biofilm. Similarly, the density of *C. albicans* also decreased (5.88 µm^3^/µm^2^, SD = 3.5) after thymol treatment (Figure 5G).

In addition, the roughness coefficients (Ra*) of biofilms developed with *C. albicans* and incubated with xanthohumol (2.34, SD = 0.47) and thymol (2.58, SD = 0.39) were significantly higher than those grown in the absence of the extracts (2.04 SD = 0.22) and similar to those developed without the fungus (2.47, SD = 0.72).

### 3.5. Quantitative and Qualitative Analysis by qPCR

**Figure 7:**
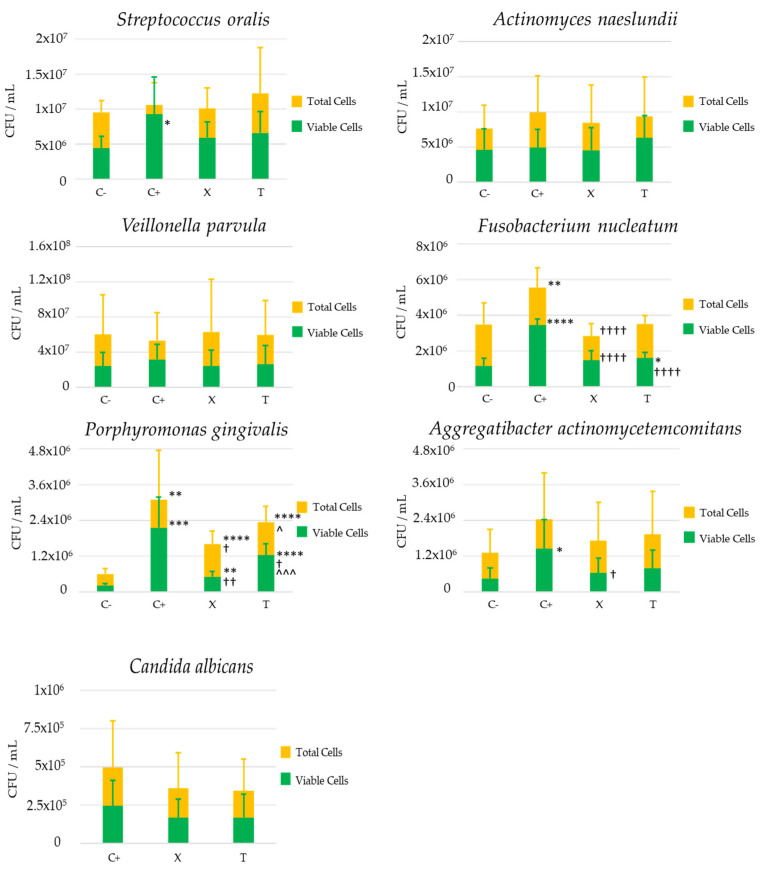
Amounts (expressed as mean and standard deviation (SD) of total and live microbial species (colony-forming units (CFUs)/mL)) determined by quantitative polymerase chain reaction (qPCR) in control negative biofilms developed without *C. albicans* (C−), positive control mixed biofilms developed with *C. albicans* (C+), mixed biofilms developed with *C. albicans* and incubated with xanthohumol (X) and mixed biofilms developed with *C. albicans* and incubated with thymol (T) (*n* = 9), using specific primers and probes directed to the 16S rRNA gene of bacterial strains and ITS2 region of *C. albicans*. * *p* < 0.05, ** *p* < 0.01, *** *p* < 0.005 and **** *p* < 0.001: statistically significant differences when comparing negative control biofilms (C−) with positive control biofilms (C+) or mixed biofilms incubated with xanthohumol (X) or thymol (T). † *p* < 0.05, †† *p* < 0.01 and †††† *p* < 0.001: statistically significant differences when comparing positive control biofilms (C+) whit biofilms incubated with xanthohumol (X) or thymol (T). ^ *p* < 0.05 and ^^^ *p* < 0.005: statistically significant differences when comparing between biofilms incubated with xanthohumol (X) and thymol (T). Comparisons between groups were performed considering viable cells and total cells. Figure corresponding to Supplementary Table S1.

Biofilms developed in the presence of *C. albicans* demonstrated significantly higher counts of *F. nucleatum* and *P. gingivalis*, a pattern also observed to a lesser extent on *A. actinomycetemcomitans*. Similarly, the cell viability of *S. oralis*, *F. nucleatum*, *P. gingivalis* and *A. actinomycetemcomitans* was also significantly higher than in biofilms grown without the fungus (Figure 7 and Table S1).

The mixed biofilm treatment with xanthohumol significantly decreased the amount of *F. nucleatum* and *P. gingivalis*. Cell viability of *F. nucleatum*, *P. gingivalis*, and *A. actinomycetemcomitans* was also significantly reduced in the same biofilms incubated with xanthohumol. The viability ratio of these bacteria was similar to that obtained in biofilms developed without the fungus and treatment. This effect was observed to a more limited extent for *S. oralis*.

The impact of thymol on biofilms with *C. albicans* resulted in lower counts of *P. gingivalis* and lower cell viability of *F. nucleatum* and *P. gingivalis*, reductions that were statistically significant when compared to the untreated mixed biofilms. No effect of thymol was observed on *A. actinomycetemcomitans* and the effect on *P. gingivalis* was significantly reduced compared to that exerted by xanthohumol.

The treatments with both substances had no quantitative impact on *C. albicans*, *A. naeslundii*, and *V. parvula*.

## 4. Discussion

The results of the present in vitro study have clearly shown that the treatment with phytochemicals, mainly xanthohumol and, to a lesser extent, thymol, inhibited the development of pseudohyphae during the biofilm growth of *C. albicans*, which significantly influenced the maturation of a validated in vitro dynamic multispecies biofilm model using periodontopathogenic bacterial strains developed on dental implant surfaces. These treatments impacted the biofilm size and the cell viability of *F. nucleatum, P. gingivalis*, *A. actinomycetemcomitans*, and *S. oralis* bacterial strains.

The SEM images (Figure 4) revealed the positive interaction between the *C. albicans* filaments and the biofilm-forming bacteria, resulting in larger and well-structured biofilm architectures. In fact, the hyphal formation favored by the conditions of the peri-implant sulcus/pocket (37 °C, presence of Gram-negative bacteria, and anaerobic environment) is the main virulence factor of *C. albicans* [24] and these filamentous forms are associated with the secretion of hydrolytic enzymes such as proteases, lipases, and hemolysins and the production of inflammatory cytokines that favor the unresolved chronic inflammatory condition [20,48]. Furthermore, the presence of *C. albicans* has been linked to the etiopathology of peri-implant diseases through their dysbiotic effect by increasing the quantity and cell viability of periodontopathogenic bacteria [25] and generating positive inter-microbial aggregates and interactions (Figure 5 and Figure 7).

The treatment with phytochemicals, namely xanthohumol, prevented these complexes’ formation during the biofilm maturation on the implant surfaces (Figure 4). This antifungal activity is based on the inhibition of mycelial growth and spore germination by interfering with the regulation of cell wall synthesis and cell wall stability [49]. This study also reports the additional ability of xanthohumol to inhibit the morphological transition from yeast to filamentous form in *C. albicans*, both in pure cultures and mixed biofilms (Figure 4). It should be noted that the application of xanthohumol doses below the MIC did not affect fungal viability (Figure 3 and Figure 7), but impacted the filamentation regulation, probably mediated by a transcriptional regulatory network (TRN) leading to the inhibition of the Ras1-Cyr1/cAMP-PKA pathway, which was involved in hyphal synthesis [50]. TRN comprises nine transcription factors with prion-binding domains (PrLDs) at the *ROB1^9465^* allele [51,52]. The inhibition of the filamentous growth in the xanthohumol-incubated biofilms led to smaller biofilms (Figure 5), probably by diminishing the bacterial adhesion and migratory motility mediated by the *C. albicans* hyphae [11]. In addition, xanthohumol has a potential inhibitory effect on quorum-sensing, which may decrease the accumulation of extracellular polymeric substances (EPSs) generated by *C. albicans* within the biofilm matrix, thus resulting in biofilms with a less complex architecture [53].

Similarly, the treated complex biofilms, compared with untreated controls, resulted in significant reductions in the viability of *F. nucleatum, P. gingivalis, A. actinomycetemcomitans*, and, to a lesser extent, *S. oralis* (Figure 7 and Table S1). Thus, the increase in these bacteria reported with biofilms grown in the presence of *C. albicans* was partially or completely reversed when the biofilm was incubated with xanthohumol. By inhibiting the filamentous growth, there could be a reduced interaction between the fungal cell wall protein FLO9 and the bacterial adhesin radD of *F. nucleatum*, thus affecting their cell survival and, consequently, limiting the mechanical bridging role that *C. albicans* may play between primary colonizers and late colonizers, such as *P. gingivalis* and *A. actinomycetemcomitans* [29,30,54]. Similarly, the absence of *C. albicans* filaments may prevent the formation of the anaerobic environments generated by the hyphae, thus limiting the viability of *P. gingivalis* and *A. actinomycetemcomitans* [27,55]. Along the same lines, the interlin InlJ, involved in the expression of *P. gingivalis* genes related to the interaction with *C. albicans* hyphae, and the adhesins Als3, associated with the interaction with the fungal Sap6 and Sap 9 proteases, may have their activity reduced, decreasing the aggregation of the bacteria [26,55]. These molecular interactions may also occur at the level of the initial colonizers, although the impact of xanthohumol was lesser on *S. oralis*. A possible mechanism may be the inhibition of the specific, dual, affinity-based interaction between the fungal adhesins Als1, Als2, Als3, and Hwp1 and the cell wall receptors SspA and SspB [56], which may be related to the smaller overall biofilm size observed by CLSM. Furthermore, the inhibition of filament formation may also reduce the interactions between cocci and the gtfR glucan-binding domain in the *C. albicans* cell wall [57].

Thymol also inhibited *C. albicans* filamentous growth similar to xanthohumol (Figure 3 and Figure 4) through the interaction with the enzymatic activities involved in hyphal cell wall formation [36,37]. However, the overall impact of thymol on the size and viability of multispecies biofilms developed with *C. albicans* was notably less than that observed with xanthohumol (Figure 5). In fact, when these mixed biofilms were incubated with thymol, it only affected the vitality of *F. nucleatum* and *P. gingivalis*, and always to a lesser extent compared with xanthohumol (Figure 7 and Table S1). This is depicted in Figure 6, with mixed biofilms incubated with xanthohumol having a clear lesser overall biomass than biofilms developed in thymol.

In the present work, the use of sublethal concentrations of xanthohumol and thymol, which did not influence the growth of the bacterial strains, prevented the filamentous development of *C. albicans*, which resulted in a decrease in the size and viability of the resulting biofilms. Thus, the ratio of viable anaerobic periodontopathogenic bacteria in the biofilm decreased with the treatments. In addition, the roughness of the biofilms incubated in the presence of the extracts increased significantly, compared to the control biofilms.

In a global context where resistance to antibiotic and antifungal agents is increasing [58], the results of the present study indicate the potential of phytochemicals as an alternative in the prevention and treatment of peri-implant and periodontal diseases. Indeed, thymol and xanthohumol, applied at appropriate concentrations, have shown a strong bactericidal effect against *F. nucleatum, P. gingivalis, A. actinomycetemcomitans, Tannerella forsythia, Campylobacter rectus* and *Treponema denticola* [33,34], against multispecies oral biofilms containing *C. albicans* [59], against *S. mutans* [60] and against multispecies biofilms formed by the same bacterial strains used in this study [35].

Some limitations of this in vitro study should also be noted: (1) the in vitro biofilm model used is intended to represent the biological diversity of the oral cavity, but natural supramarginal and subgingival biofilms consist of hundreds of microbial species; (2) although the used dynamic biofilm model mimics the oral cavity conditions, there are variables that cannot be reproduced, including different behaviors and immune responses of patients, what makes it difficult to replicate in vivo the conditions of this in vitro research; (3) working with live microorganisms is associated with a higher than desirable variability, which should be considered when interpreting the results, especially in the biofilms incubated with thymol.

However, the qualitative and quantitative results reported in the present study, as well as their statistical processing, suggest that the exacerbation of the quantity and viability of the periodontopathogenic bacteria *F. nucleatum, P. gingivalis*, and *A. actinomycetemcomitans*, in biofilms developed in the presence of *C. albicans*, is due to the filamentous growth of the fungus and that this effect can be controlled by inhibiting the formation of these fungal hyphae. These findings highlight the possibility of using therapies with dual action, being antimicrobial at the appropriate lethal concentrations and, at the same time, interfering with critical biofilm intermicrobial interactions.

In conclusion, xanthohumol and, to a lesser extent, thymol have been shown to inhibit, in vitro, the pseudohyphal growth and filamentation of *C. albicans*, thus reverting the dysbiotic effect that *C. albicans* exerts on the growth of complex biofilms on implant surfaces.

## Data Availability

Data are contained within the article.

## Supplementary Materials

The following supporting information can be downloaded at https://www.mdpi.com/article/10.3390/cells13221877/s1: Table S1: Counts (expressed as mean and standard deviation (SD) and 95% confidence intervals (CIs)) of total and live microbial species (colony-forming units (CFUs)/mL) determined by quantitative polymerase chain reaction (qPCR) in control negative biofilms, developed without *Candida albicans* (C−), positive control mixed biofilms, developed with *C. albicans* (C+), mixed biofilms developed with *C. albicans* and incubated with xanthohumol (X) and mixed biofilms developed with *C. albicans* and incubated with thymol (T) (*n* = 9), using specific primers and probes directed to the 16S rRNA gene of bacterial strains and ITS2 region of *C. albicans*.
