# Supplementary material for: The Effect of Xanthohumol and Thymol on Candida albicans Filamentation and Its Impact on the Structure, Size, and Cell Viability of Biofilms Developed over Implant Surfaces
Source: Cells. 2024 Nov 13;13(22):1877. doi: 10.3390/cells13221877 (PMC11593281; doi:10.3390/cells13221877)
Supplement: Supplementary file 1 [file cells-13-01877-s001.zip › cells-3268608-supplementary.pdf]

Supplementary Table S1. Counts (expressed as mean and standard deviation (SD) and 95% confidence intervals (CI) of total and live microbial species (colony-forming units (CFUs)/mL) determined by quantitative polymerase chain reaction (qPCR) in control negative biofilms, developed without *Candida albicans* (C-), positive control mixed biofilms, developed with *C. albicans* (C+), mixed biofilms developed with *C. albicans* and incubated with xanthohumol (X), and mixed biofilms developed with *C. albicans* and incubated with thymol (T) (n=9), using specific primers and probes directed to the 16S rRNA gene of bacterial strains and ITS2 region of *C. albicans*. * $p < 0.05$, ** $p < 0.01$, *** $p < 0.005$ and **** $p < 0.001$: statistically significant differences when comparing negative control biofilms (C-) with positive control biofilms (C+) or mixed biofilms incubated with xanthohumol (X) or thymol (T). † $p < 0.05$, †† $p < 0.01$ and †††† $p < 0.001$: statistically significant differences when comparing positive control biofilms (C+) with biofilms incubated with xanthohumol (X) or thymol (T). ^ $p < 0.05$, ^^ $p < 0.01$ and ^^^ $p < 0.005$: statistically significant differences when comparing between biofilms incubated with xanthohumol (X) and thymol (T). Comparisons between groups were performed considering viable cells and total cells.

Species	Analysis	Condition	Microbial counts (CFU/ml)				% Viability	
					95%CI			
			Mean	(SD)	Lower limit	Upper limit	Mean	(SD)
Streptococcus oralis	Total	C-	9,57x10^{6}	1,64x10^{6}	6,09x10^{6}	1,21x10^{7}		
		C+	1,06x10^{7}	3,22x10^{6}	4,47x10^{6}	1,48x10^{7}		
		X	1,01x10^{7}	2,96x10^{6}	3,69x10^{6}	1,21x10^{7}		
		T	1,23x10^{7}	6,54x10^{6}	2,89x10^{6}	2,77x10^{7}		
	Viable	C-	4,44x10^{6}	1,65x10^{6}	2,21x10^{6}	6,37x10^{6}	46,85	16,81
		C+	9,29x10^{6} *	5,33x10^{6}	4,56x10^{5}	1,91x10^{7}	80,61 *	34,95
		X	5,95x10^{6}	2,26x10^{6}	7,88x10^{4}	7,43x10^{6}	56,38	23,66
		T	6,58x106	3,10x10^{6}	9,32x10^{4}	1,13x10^{7}	55,11	29,55
Actinomyces naeslundii	Total	C-	7,66x10^{5}	3,32x10^{5}	3,74x10^{5}	1,45x10^{6}		
		C+	9,93x10^{5}	5,22x10^{5}	3,17x10^{5}	1,65x10^{6}		
		X	8,44x10^{5}	5,41x10^{5}	2,32x10^{5}	1,80x10^{6}		
		T	9,39x10^{5}	5,62x10^{5}	3,45x10^{4}	1,78x10^{6}		
	Viable	C-	4,63x10^{5}	2,94x10^{5}	1,72x10^{5}	8,71x10^{5}	56,88	19,06
		C+	4,93x10^{5}	2,58x10^{5}	1,82x10^{5}	9,38x10^{5}	56,38	21,94
		X	4,55x10^{5}	3,19x10^{5}	7,75x10^{4}	1,02x10^{6}	55,16	20,76
		T	6,33x10^{5}	3,19x10^{5}	1,59x10^{5}	1,21x10^{6}	47,23	15,93
Veillonella parvala	Total	C-	6,03x10^{7}	4,52x10^{7}	5,15x10^{6}	1,19x10^{8}		
		C+	5,36x10^{7}	3,18x10^{7}	4,84x10^{6}	9,62x10^{7}		
		X	6,33x10^{7}	5,98x10^{7}	1,05x10^{7}	1,11x10^{8}		
		T	5,98x10^{7}	3,90x10^{7}	5,10x10^{6}	1,25x10^{8}		
	Viable	C-	2,42x10^{7}	1,58x10^{7}	3,49x10^{6}	3,83x10^{7}	52,43	19,89
		C+	3,17x10^{7}	1,73x10^{7}	3,16x10^{6}	4,95x10^{7}	62,5	12,13
		X	2,43x10^{7}	1,81x10^{7}	5,06x10^{6}	5,00x10^{7}	38,92 †	17,94
		T	2,63x10^{7}	2,11x10^{7}	4,20x10^{6}	5,95x10^{7}	59,02	27,65

Fusobacterium nucleatum	Total	C-	$3,50x10^{6}$	$1,18x10^{6}$	$2,15x10^{6}$	$5,17x10^{6}$		
		C+	$5,56x10^{6}$ **	$1,10x10^{6}$	$4,22x10^{6}$	$6,94x10^{6}$		
		X	$2,83x10^{6}$ ††††	$6,96x10^{5}$	$1,45x10^{6}$	$3,66x10^{6}$		
		T	$3,53x10^{6}$	$4,53x10^{5}$	$2,81x10^{6}$	$4,20x10^{6}$		
	Viable	C-	$1,16x10^{6}$	$4,23x10^{5}$	$1,62x10^{5}$	$1,64x10^{6}$	37,20	7,90
		C+	$3,45x10^{6}$ ****	$3,57x10^{5}$	$2,92x10^{6}$	$3,90x10^{6}$	63,81****	11,99
		X	$1,47x10^{6}$ ††††	$5,22x10^{5}$	$5,47x10^{5}$	$2,31x10^{6}$	46,66 ††	12,13
		T	1,62x10 * ††††	$3,11x10^{5}$	$1,30x10^{6}$	$2,14x10^{6}$	48,39** ††	7,07
Porphyromonas gingivalis	Total	C-	$6,10x10^{5}$	$1,78x10^{5}$	$4,05x10^{5}$	$9,28x10^{5}$		
		C+	$3,09x10^{6}$ **	$1,67x10^{6}$	$8,32x10^{5}$	$6,82x10^{6}$		
		X	$1,61x10^{6}$ **** †	$4,32x10^{5}$	$7,84x10^{5}$	$2,08x10^{6}$		
		T	$2,35x10^{6}$ **** ^	$5,28x10^{5}$	$1,16x10^{6}$	$2,88x10^{6}$		
	Viable	C-	$2,14x10^{5}$	$5,98x10^{4}$	$1,36x10^{5}$	$2,92x10^{5}$	35,57	5,62
		C+	$2,15x10^{6}$ ***	$1,03x10^{6}$	$3,60x10^{5}$	$4,01x10^{6}$	69,27 ****	13,13
		X	$5,08x10^{5}$ ** ††	$1,84x10^{5}$	$1,96x10^{5}$	$8,51x10^{5}$	32,28 ††††	10,92
		T	$1,24x10^{6}$ **** † ^^^	$3,87x10^{5}$	$6,74x10^{5}$	$1,61x10^{6}$	53,69* † ^^	14,66
Aggregatibacter actinomycetemcomitans	Total	C-	$1,32x10^{6}$	$7,91x10^{5}$	3,39X105	2,42X106		
		C+	$2,44x10^{6}$	$1,56x10^{6}$	6,11X105	4,51X106		
		X	$1,73x10^{6}$	$1,28x10^{6}$	4,78X105	4,25X106		
		T	$1,93x10^{6}$	$1,45x10^{6}$	7,10X105	4,81X106		
	Viable	C-	$4,62x10^{5}$	$3,46x10^{5}$	$1,19x10^{5}$	$1,09x10^{6}$	36,52	14,28
		C+	$1,46x10^{6}$ *	$9,83x10^{5}$	$2,72x10^{5}$	$2,72x10^{6}$	69,97 **	27,41
		X	$6,47x10^{5}$ †	$4,87x10^{5}$	$1,68x10^{4}$	$1,49x10^{6}$	36,23 ††	14,35
		T	$8,01x10^{5}$	$6,19x10^{5}$	$6,84x10^{4}$	$1,94x10^{6}$	41,22	17,94
Candida albicans	Total	C+	$4,96x10^{5}$	$3,06x10^{5}$	$5,96x10^{4}$	$9,09x10^{5}$		
		X	$3,59x10^{5}$	$2,35x10^{5}$	$5,55x10^{4}$	$7,37x10^{5}$		
		T	$3,43x10^{5}$	$2,09x10^{5}$	$8,17x10^{4}$	$5,38x10^{5}$		
	Viable	C+	$2,45x10^{5}$	$1,68x10^{5}$	$4,94x10^{4}$	$4,63x10^{5}$	50,63	16,48
		X	$1,67x10^{5}$	$1,22x10^{5}$	$6,36x10^{3}$	$3,14x10^{5}$	53,5	21,65
		T	$1,68x10^{5}$	$1,53x10^{5}$	$7,65x10^{3}$	$3,77x10^{5}$	38,12	19,93
